# Inflammatory fibroid polyp causing ileo-ileal intussusception

**DOI:** 10.11604/pamj.2022.42.36.35280

**Published:** 2022-05-16

**Authors:** Tarik Souiki, Khalid Mazaz

**Affiliations:** 1 Faculty of Medicine and Pharmacy, Sidi Mohammed Ben Abdellah University, Fez, Morocco,; 2 Department of Visceral Surgery, University Hospital Hassan II, Fez, Morocco

**Keywords:** Inflammatory fibroid polyp, intestinal intussusception, Vanek’s tumor

## Image in medicine

Inflammatory fibroid polyp (IFP) or Vanek's tumor is a rare and idiopathic pseudotumor arising from the submucosa of the gastrointestinal tract. The underlying cause of IFP remains unclear. Many factors have been incriminated such as intestinal trauma or eosinophilic gastroenteritis. Small bowel involvement is the second most common location of IFP after the stomach. Intestinal IFP is most often revealed by intussusception or obstruction. The mainstay treatment is surgical, which consisted of a segmental intestinal resection. The histological appearance may be a differential diagnosis with other spindle cell lesions, especially with stromal tumors. The definitive diagnosis is confirmed by immunochemistry examination. Herein, we report the clinical case of a 62-year-old female, with no notable medical history, who presented with nausea and vomiting associated with abdominal acute pain and distension evolving for about seven days. The physical examination showed a heart rate of 90 beats per minute, blood pressure of 120/70 mmHg, and body temperature of 37.5°C. The abdominal examination revealed a slightly tympanic abdomen with diffuse tenderness. Computed tomography (CT) scan of the abdomen revealed dilated small bowel loops proximally of an ileo-ileal intussusception. The wall of the invaginated loop shows an irregular, symmetrical, and short parietal thickening, enhanced after contrast and measuring 36 x 24 mm. The patient underwent an urgent midline laparotomy. Surgical exploration revealed an ileo-ileal intussusception, localized at 70 cm proximal to the ileocecal valve and causing dilatation in the intestine proximal to the intussusception. Manual disinvagination revealed intraluminal polypoid mass (star) obstructing the lumen with parietal umbilication. Segmental resection of the intussuscepted ileum and side-to-side stapler ileal anastomosis were performed. The patient had an uneventful recovery. Histological findings showed bland spindled cells, small vessels, and eosinophil-rich mixed inflammatory infiltrates. Immunohistochemical staining was positive for CD34 and negative for CD 117, DOG1 and S-100 protein. Therefore, a final diagnosis of an inflammatory fibroid polyp was made.

**Figure 1:**
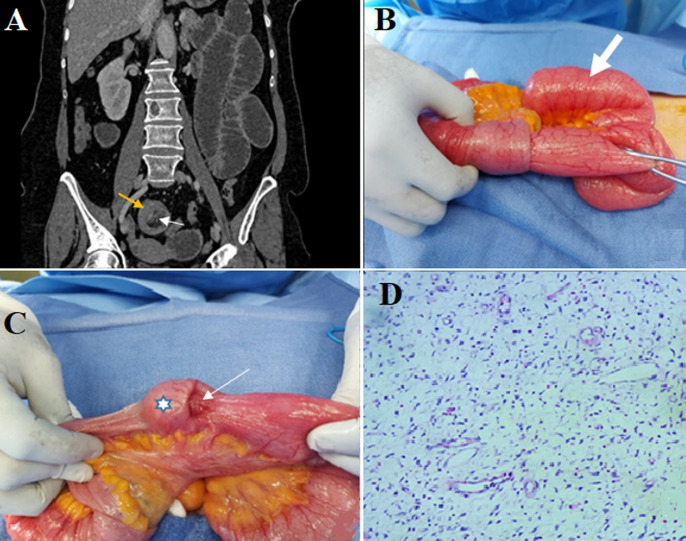
A) abdominal computed tomography scan (coronal section), showing an image of ileo-ileal intussusception with invaginated mesenteric fat (white arrow); the wall of the invaginated loop shows an irregular enhanced thickening (yellow arrow); B) per-operative view showing ileo-ileal invagination (white arrow) with dilated intestinal loops (white arrow); C) per-operative view after disinvagination showing intraluminal polypoid mass (star) with parietal umbilication (white arrow); D) histological findings showing bland spindled cells, small vessels, and eosinophil-rich mixed inflammatory infiltrate (HES x 200)

